# Editorial: Hearing Loss: From Pathogenesis to Treatment

**DOI:** 10.3389/fncel.2022.907483

**Published:** 2022-06-10

**Authors:** Zuhong He, Weijia Kong, Shengyu Zou, Hai Huang, Suhua Sha

**Affiliations:** ^1^Department of Otorhinolaryngology-Head and Neck Surgery, Zhongnan Hospital of Wuhan University, Wuhan, China; ^2^Department of Cell and Molecular Biology, Brain Institute, Tulane University, New Orleans, LA, United States; ^3^Department of Otorhinolaryngology, Union Hospital, Tongji Medical College, Huazhong University of Science and Technology, Wuhan, China; ^4^Department of Pathology and Laboratory Medicine, Medical University of South Carolina, Charleston, SC, United States

**Keywords:** sensorineural deafness, hearing loss, hair cell, neuron, auditory organ

Damage to the auditory system is associated with many factors, including genetic deficiency, aging, ototoxic drugs, noise, infection, and many other environmental factors. The mammalian inner ear is a highly differentiated organ, particularly the hair cells that cannot regenerate. Therefore, excessive damage to the inner ear cells will lead to permanent hearing loss. At present, there are limited clinical approaches to prevent and treat hearing loss due to the unique nature of the inner ear structure and cells. With the advance of new technologies and methods, including gene therapy, stem cell therapy, biomaterials, and tissue engineering, we hope that future studies will provide theoretical and experimental bases for the prevention and treatment of sensorineural hearing loss and that these basic research findings can be translated into therapeutic applications. This Frontiers Research Topic, entitled *Hearing Loss: From Pathogenesis to Treatment*, encompasses 18 contributions about the mechanisms and prevention of auditory organ injury, new technologies and biomaterials to recover hearing loss in animals and patients, and a range of promising approaches for hearing loss treatment.

The regeneration of inner ear hair cells is a hot topic arousing general interest in otology research. In this review, some important regeneration-related signal pathways have been discussed by Xu and Yang. They introduced some reports about the role of adeno-associated virus (AAV) vectors in improving hearing and summarized the role of transcription factors and epigenetic regulation in hair cell regeneration. Chai et al. have investigated the role of the super elongation complex (SEC) and its three key components in the regulation of inner ear progenitor cell differentiation. The three key components of the SEC can be detected in cochlear hair cells and supporting cells in neonatal mice, and inhibiting the activity of the SEC affects the proliferation ability of Lgr5^+^ progenitors but not the differentiation ability. Qian et al. have used zebrafish to study dual-specificity phosphatase 14 (DUSP14)-regulated hair cell fate. They found that *dusp14* knockdown will reduce the number of hair cells, neuromasts, and supporting cells, eventually causing hearing defects. RNA sequencing was performed to explore the molecular mechanism responsible for regulating the *dusp14* gene on hearing function. They found that the p38 signaling pathway plays a vital role in *dusp14* knockdown-related hearing defects, and p38 inhibitor treatment could reverse *dusp14* knockdown-induced absence of supporting cells and proliferation disability.

As commonly used antibacterial drugs, the relationship between aminoglycoside antibiotics and hearing loss has not been fully elucidated. Gao et al. have explored the protective role of monosialotetrahexosylganglioside (GM1) in aminoglycoside-induced hair cell injury. They found that GM1 could reduce the ROS level by regulating the expression balance of oxidative and antioxidant genes. GM1 also reduces the rate of apoptosis and hair cell death caused by aminoglycoside antibiotics.

The role of autophagy in the auditory system has received much attention in recent years. Zheng et al. have found that otitis media (OM) can impair the autophagy pathway in middle ear (ME) tissues. The initial stage of autophagy in OM mice was activated, but downregulation of Rab7 and Syntaxin 17 disrupts the fusion of autophagosomes and lysosomes in OM mice, thereby blocking autophagic flux. Rapamycin treatment was found to protect hearing by inhibiting the activity of mTOR1 and activating autophagy flux to reduce the inflammatory infiltrates and TNF-α expression. Yang et al. have reviewed the role of autophagy in sensorineural hearing loss (SNHL), including noise, ototoxic drugs, and age-related hearing loss. They also introduced the pro-apoptotic effect of autophagy in cisplatin-induced HEI-OC1 cell death. This review concluded the role of TFEB, PTEN, FoxG1, and STAT proteins and miRNAs in the pathogenesis of SNHL by regulating the autophagy pathway.

Xu et al. have reviewed current developments in promising nanocarrier systems for inner ear disease therapy. Hydrogel delivery systems have the advantage of enabling a higher residence time for drugs so that they reach equilibrium in the inner ear and easy degradation with good biocompatibility. As a novel drug delivery system, nanoparticle-based systems, such as nanosized polymers, peptides, silicas, and metal–organic frameworks, have been widely employed as drug delivery systems for inner ear disease therapy. Those nanoparticles could prolong the half-life of drugs and increase the solubility of drugs for ease of crossing physiological barriers. Tang et al. have discussed the role of superparamagnetic iron oxide nanoparticles (SPIOs), a biomaterial with excellent biocompatibility, in neural stem cell (NSC) proliferation. They found that SPIOs could promote NSC proliferation in the absence of a static magnetic field (SMF). However, when the SMF intensity (over 100 ± 10 mT) and SPIO concentration (more than 500 μg/ml) increased, the proliferation of NSC was suppressed. Uncovering the underlying mechanism could have profound significance for tissue engineering and regenerative medicine for SPIO applications. Ding et al. have reviewed novel materials for auditory disease treatment, including conductive and sensorineural hearing loss and auditory-related conditions. New materials used in clinical otologic surgery have the advantages of excellent biocompatibility, lower incidence of postoperative complications, and shorter postoperative recovery time. Novel drug delivery systems exhibit stable and sustained drug release, capable of transporting the drug into target cells with high selectivity.

Lu et al. have found that the systemic administration of mitochondria-target antioxidant mito-TEMPO (MT) mitigated oxidative stress in the cochlea after noise exposure, reduced the hair cell and ribbon synapse loss induced by acoustic trauma, and overall exhibited a protective effect on hearing. MT treatment could restore mtDNA and ATP levels to improve mitochondrial function during noise exposure. Furthermore, they found that MT treatment attenuated TFAM and SOD2 expression reduction after noise exposure independent of the PGC-1α/NRF-1/TFAM pathway. In addition, MT treatment partially recovered the TFAM–mtDNA interaction and reduced the amount of naked mtDNA in the cytoplasm of outer hair cells. Zhang et al. used desmopressin injections to generate an endolymphatic hydrops (EH) model to mimic Meniere's disease. They observed the cochlear structure and hearing changes in the EH model. The increased hearing loss and reduced round window membrane vibration were found to correlate positively with the severity of EH. Then, a mannitol–saline solution was used as a dehydration treatment to recover hearing in the EH model, showing that rehydration can recover the early hearing loss in ER but not after a long duration.

Yang et al. have focused on the role of histidyl-tRNA synthetase HARS2 in cochlear hair cell development. They found that *Hars2* conditional knockout mice exhibited gradual hearing loss at P30 and complete hearing loss at P60. The abnormal mitochondria began to appear in HCs at P14 of the *Hars2* CKO mice, which indicated reduced inner membrane surface and low-density mitochondrial mass. As a result, the expression of the antioxidant genes xCT, Nqo1, Sod2, and Gsr significantly decreased, and apoptosis HCs gradually appeared at P45. They confirmed that *Hars2* is a critical gene for hair cell survival and maintaining the normal function of mitochondria. *GJB2* gene mutations were the predominant cause of hereditary deafness. However, current research focuses on the role of the *GJB2* gene in the morphological development of the organ of Corti. Yang et al. have reported the role of *GJB2* in Kölliker's organ. *Gjb*2^loxP/loxP^ and ROSA26^CreER^ mice were used in this study, and TUNNEL-positive cells were detected in Kölliker's organ at P1. Meanwhile, autophagy flux was also blocked in Cx26-cKD mice. The Cx26 knockdown will reduce the ATP level in lysosomal vesicles and affect the ATP-dependent Ca^2+^ signaling pathway in Kölliker's organ, which is crucial for hearing acquisition. Yang et al. also used CRISPR/Cas9 technology to generate connexin 30 (Cx30) knockout mice and confirmed the important role of Cx30 in hearing development. Compared with WT mice, Cx30-deficient mice exhibited mild hearing loss from 4 to 16 kHz at 6 months. The expression of the Cx26 protein was decreased in the cochlea of Cx30 knockout mice. The structure of the stria vascularis, endocochlear potential, and ATP release were affected in Cx30 knockout mice.

Li et al. have established a “trinomial forced-choice method” to match tinnitus pitch with 16 frequency measurement points from 250 Hz to 16 kHz. They found that clinical patients with normal hearing were almost within the high-frequency range of tinnitus pitch. They have demonstrated a more direct correspondence between tinnitus pitch and hearing frequency deficits.

In hearing protection-related research, the damage mechanism of cochlear hair cells has always been the focus of attention. Sun et al. have reviewed the damage mechanism of spiral ganglion neurons (SGNs) in different hearing injury models. They have also introduced some potential therapies to reduce SGN damage. Adenovirus (Ad)-based and AAV-based vectors mediated exogenous NT (BDNF, GDNF, NT3, CNTF, and others) and provided good results in SGN protection. Transplanted embryonic stem cells were successfully engrafted into the modiolus and formed ectopic ganglia with differentiated neuronal-type cells. However, many challenges must be overcome before applications can be made for humans. Liu et al. have explored the function of glutathione peroxidase 1 (GPX1) on cochlear SGNs in *gpx1* knockout mice (*gpx*1^−/−^) after peroxynitrite treatment. They found that after culturing with peroxynitrite, GPX1 expression significantly decreased in cochlear SGNs. In *gpx*1^−/−^ mice, the cell-counting results showed that SGNs experienced severe damage after peroxynitrite treatment compared with WT mice. Ebselen pretreatment, a GPX1 mimic, can reverse peroxynitrite-induced SGN apoptosis and cell loss. Furthermore, they found that NF-κB pathway activation plays an important role in peroxynitrite-induced SGN damage. NF-κB inhibitors and upregulating GPX1 expression can block NF-κB pathway activation through downstream pro-apoptosis–related gene expression.

Sun et al. have performed a cross-sectional population-based study to analyze the link between hearing impairment and air pollution. After adjusting for confounding factors, they found that exposure to PM_10_, NO_X_, and NO_2_ was linked to hearing impairment. However, PM_2.5_ and PM_2.5_ absorbance showed no association with hearing loss. They proposed that oxidative stress and pro-inflammatory pathways may be involved in the association between air pollution and hearing loss.

In conclusion, the research articles and reviews collected in this Research Topic provide a comprehensive set of information on the damage mechanisms and protection methods of hair cells and SGN, hair cell regeneration, and the application of new materials and approaches in the treatment of hearing diseases. Together, the achievements included in this Research Topic contribute to further understanding of the pathogenesis of hearing loss, attracting the interest of readers in hearing-related research, and providing a reference for the development of novel hearing loss therapies.

## Author Contributions

WK, SZ, and ZH participated in writing the manuscript. ZH, SZ, WK, HH, and SS participated in reviewing the manuscript. All authors contributed to the article and approved the submitted version.

## Conflict of Interest

The authors declare that the research was conducted in the absence of any commercial or financial relationships that could be construed as a potential conflict of interest.

## Publisher's Note

All claims expressed in this article are solely those of the authors and do not necessarily represent those of their affiliated organizations, or those of the publisher, the editors and the reviewers. Any product that may be evaluated in this article, or claim that may be made by its manufacturer, is not guaranteed or endorsed by the publisher.

